# US Food and Drug Administration Review Time of Supplemental New Indication Approvals of Drugs and Biologics, 2017 to 2019

**DOI:** 10.1001/jamanetworkopen.2023.18889

**Published:** 2023-06-26

**Authors:** Meera M. Dhodapkar, Joseph S. Ross, Reshma Ramachandran

**Affiliations:** 1 Yale School of Medicine, Yale University, New Haven, Connecticut; 2 Section of General Medicine, Department of Internal Medicine, Yale School of Medicine, New Haven, Connecticut; 3 Department of Health Policy and Management, Yale School of Public Health, New Haven, Connecticut; 4 Yale Collaboration for Regulatory Rigor, Integrity, and Transparency, Yale School of Medicine, New Haven, Connecticut; 5 Center for Outcomes Research and Evaluation, Yale-New Haven Health System, New Haven, Connecticut

## Abstract

This cross-sectional study examines US Food and Drug Administration regulatory review time of supplemental new indication approvals of drugs and biologics between 2017 and 2019.

## Introduction

In October 2022, the US Food and Drug Administration (FDA) announced the Split Real-Time Application Review (STAR) pilot program under the Prescription Drug User Fee Authorization Act VII.^1^ Like its predecessor, the Real-Time Oncology Review (RTOR) program for oncology drugs, STAR is designed to shorten regulatory review of supplemental new indication approvals of drugs and biologics that treat serious conditions with an unmet need. The STAR program allows the FDA to begin reviewing Part 1 submissions (containing all components of the efficacy supplement except for final clinical study reports and integrated summaries of effectiveness and safety) as received, in a split fashion, with Part 2 submissions (containing the aforementioned items) submitted roughly 2 months later.

Preliminary evidence suggests that supplemental new indication applications were reviewed 3 months faster within RTOR compared with those only designated for priority review.^2,3^ However, baseline regulatory review times for STAR for nononcology indications are not established. Accordingly, we characterized the FDA regulatory review time of supplemental new indication approvals of drugs and biologics approved between 2017 and 2019.

## Methods

From a previously collected sample of all new supplemental indication approvals of drugs and biologics between January 1, 2017, and December 31, 2019,^4^ we identified submission and approval dates from approval letters obtained from the Drugs@FDA database. We used previously described methods to determine whether supplemental new indications were approved under other special regulatory programs and their therapeutic area.^3,4^ Regulatory review times were rounded to the nearest month and described using medians and IQRs. Institutional review board approval and informed consent were not required because this cross-sectional study was based on publicly available information and involved no patient records, in accordance with 45 CFR §46. The study followed the STROBE reporting guideline. All analyses were performed using JMP Pro, version 16.0.0 (SAS Institute). Data analysis was performed between October 28 and November 11, 2022.

## Results

From 2017 to 2019, the FDA approved 107 therapeutics for 146 supplemental new indications, including 99 small molecule drugs (67.8%) and 47 biologics (32.2%) (Table). Of these, 78 (53.4%) were oncology therapeutics and 74 (50.7%) received at least 1 special regulatory review designation, including 50 (64.1%) designated for priority review and 12 (8.2%) reviewed under RTOR. Of the 50 designated for priority review, 12 (24.0%) were also approved under RTOR.

**Table.** Regulatory Review Time for US Food and Drug Administration Supplemental New Indication Approvals of Drugs and Biologics Between 2017 and 2019

Stratification category	No. of approvals (%) (N = 146)	Median regulatory review time (IQR), mo^a^	*P* value
Agent type			
Pharmacologic	99 (67.8)	6 (6-10)	.20
Biologic	47 (32.2)	6 (6-10)	
Special regulatory program			
None	72 (49.3)	10 (6-10)	<.001^b^
Any	74 (50.7)	6 (5-6)	
Accelerated approval	20 (13.7)	6 (6-6)	
Breakthrough	35 (24.0)	6 (4-6)	
Non-RTOR priority review	49 (33.6)	6 (5-6)	
RTOR	12 (8.2)	3 (2-4.5)	<.001^c^
Therapeutic area			
Oncology	78 (53.4)	6 (5-6)	<.001
Nononcology	68 (46.6)	10 (6-10)	

Abbreviation: RTOR, Real-Time Oncology Review.

^a^ Overall median regulatory review time was 6 (IQR, 6-10) months.

^b^ Represents comparison between time to approval for approvals under any special regulatory program vs none.

^c^ Represents comparison between time to approval for RTOR and non-RTOR priority review approvals.

The median regulatory review time was 6 (IQR, 6-10) months, with no difference between drugs and biologics (6 [IQR 6-10] months for both; *P* = .20) (Table). However, the median regulatory review time was significantly shorter for oncology therapeutics that received at least 1 special regulatory review designation (6 [IQR, 5-6] months) compared with nononcology therapeutics (10 [6-10] months; *P* < .001) and for oncology therapeutics reviewed under RTOR (3 [2-4.5] months) compared with non-RTOR approvals designated for priority review (6 [5-6] months; *P* < .001).

## Discussion

In this cross-sectional study of 146 supplemental new indications approved by the FDA between 2017 and 2019, the median regulatory review time was 6 months. Consistent with prior studies, the shortest review times were for supplemental new indication approvals reviewed under RTOR, which were roughly half the duration of non-RTOR oncology approvals designated for priority review.^3^ Limitations of this study include its 3-year sample and not accounting for approvals in other countries, which the FDA may have considered in its evaluations.

These findings inform efforts to launch STAR and suggest that the program may shorten regulatory review times for supplemental new indication approvals. However, the administrative burden required to meet shorter deadlines deserves consideration, particularly since physicians can prescribe approved products off label and FDA approval may not affect insurance coverage. In addition, shorter regulatory review times may be associated with unintended risks, as shorter regulatory review has been associated with an increased risk of FDA safety actions after approval for new drugs.^5,6^ The STAR program should ensure that the FDA has adequate resources to staff more rapid review of supplemental new indication applications.

## Notes

Supplement.
Data Sharing Statement
