# Supplementary material for: US Food and Drug Administration Review Time of Supplemental New Indication Approvals of Drugs and Biologics, 2017 to 2019
Source: JAMA Netw Open. 2023 Jun 26;6(6):e2318889. doi: 10.1001/jamanetworkopen.2023.18889 (PMC10293905; doi:10.1001/jamanetworkopen.2023.18889)
Supplement: Supplement. — Data Sharing Statement [file jamanetwopen-e2318889-s001.pdf]

Data Sharing Statement

Dhodapkar. US Food and Drug Administration Review Time of Supplemental New Indication Approvals of Drugs and Biologics, 2017 to 2019. *JAMA Netw Open*. Published June 26, 2023. doi:10.1001/jamanetworkopen.2023.18889

Data
Data available: No

Additional Information
Explanation for why data not available: Data for this study will be made available upon request.
